# The Beneficial Effects of Principal Polyphenols from Green Tea, Coffee, Wine, and Curry on Obesity

**DOI:** 10.3390/molecules26020453

**Published:** 2021-01-16

**Authors:** Tomokazu Ohishi, Ryuuta Fukutomi, Yutaka Shoji, Shingo Goto, Mamoru Isemura

**Affiliations:** 1 Institute of Microbial Chemistry (BIKAKEN), Numazu, Microbial Chemistry Research Foundation, Shizuoka 410-0301, Japan; 2 Quality Management Div. Higuchi Inc., Minato-ku, Tokyo 108-0075, Japan; fukutomi_ryuuta@mac.com; 3 Graduate School of Integrated Pharmaceutical and Nutritional Sciences, University of Shizuoka, Shizuoka 422-8526, Japan; yutaka_shoji@yahoo.co.jp (Y.S.); isemura@u-shizuoka-ken.ac.jp (M.I.); 4 Division of Citrus Research, Institute of Fruit Tree and Tea Science, National Agriculture and Food Research Organization (NARO), Shimizu, Shizuoka 424-0292, Japan; gotos@affrc.go.jp

**Keywords:** polyphenols, epigallocatechin-3-*O*-gallate, chlorogenic acid, resveratrol, curcumin, obesity, reactive oxygen species, 5′-AMP-activated protein kinase, nuclear factor-κB, randomized controlled trial

## Abstract

Several epidemiological studies and clinical trials have reported the beneficial effects of green tea, coffee, wine, and curry on human health, with its anti-obesity, anti-cancer, anti-diabetic, and neuroprotective properties. These effects, which have been supported using cell-based and animal studies, are mainly attributed to epigallocatechin gallate found in green tea, chlorogenic acid in coffee, resveratrol in wine, and curcumin in curry. Polyphenols are proposed to function via various mechanisms, the most important of which is related to reactive oxygen species (ROS). These polyphenols exert conflicting dual actions as anti- and pro-oxidants. Their anti-oxidative actions help scavenge ROS and downregulate nuclear factor-κB to produce favorable anti-inflammatory effects. Meanwhile, pro-oxidant actions appear to promote ROS generation leading to the activation of 5′-AMP-activated protein kinase, which modulates different enzymes and factors with health beneficial roles. Currently, it remains unclear how these polyphenols exert either pro- or anti-oxidant effects. Similarly, several human studies showed no beneficial effects of these foods, and, by extension polyphenols, on obesity. These inconsistencies may be attributed to different confounding study factors. Thus, this review provides a state-of-the-art update on these foods and their principal polyphenol components, with an assumption that it prevents obesity.

## 1. Introduction

Plant polyphenols are often found in beverages such as tea, wine, and coffee and in vegetables and fruits such as turmeric, onions, broccoli, apples, berries, citrus fruits, and plums [1].

**Figure 1:**
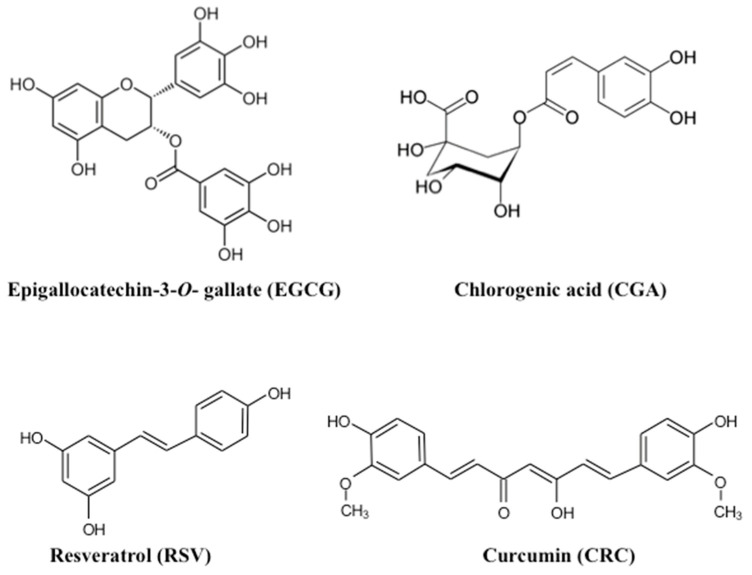
Chemical structures of epigallocatechin-3-*O*-gallate (EGCG), chlorogenic acid (CGA), resveratrol (RSV), and curcumin (CRC).

Green tea is produced from leaves of the *Camellia sinensis* plant; a cup of green tea brewed from 2.5 g tea leaves has been reported to contain 240–320 mg catechins, of which (−)-epigallocatechin-3-*O*-gallate (EGCG, Figure 1) accounts for 60%–65% [2,3]. Black tea is similarly produced from these leaves by intrinsic enzymatic processing and microorganisms during which catechins are polymerized to generate lower EGCG levels.

Coffee contains approximately 2000 different chemicals; the major polyphenols are chlorogenic acid (CGA, Figure 1) and its derivatives, which account for approximately 3% *w*/*w* of the roasted coffee powder [2,3,4]. A single cup of coffee may contain 20–675 mg CGA [3].

Resveratrol (RSV, 3,4′,5-trihydroxystilbene, Figure 1) is often derived from several plant sources, including grapes, peanuts, and berries, and it exists in two isomeric forms: cis- and trans-RSV. Trans-RSV (defined here as RSV, Figure 1) is naturally found in grape skin and in the leaf epidermis of the grape vine and is the main RSV form in red grape juice (3.38 mg/L) [1,4]. Daily RSV intake may be in the order of several mg/day [5].

Curcumin (CRC, Figure 1) is a yellow pigment, an active component of the turmeric plant (*Curcuma longa*), and is widely used in cooking, cosmetics, dyes, and medicines [1,6]. In Asian populations, approximately 100 mg CRC is consumed daily [1,4].

Several studies have shown that consuming tea, coffee, wine, and curry can be beneficial in fighting against various diseases including, cancer, obesity, neurodegenerative disorders, and diabetes. Their polyphenolic constituents are believed to contribute mainly to these effects as discussed in our previous reviews [1,2,3,6,7].

Thus, this review summarizes contemporary data from human epidemiological obesity studies on these polyphenols and discusses mechanistic aspects related to laboratory findings from cell and animal experiments.

## 2. The Effects of Green Tea/EGCG on Obesity

Epidemiological studies have suggested that green tea and its principal constituent, EGCG, exert beneficial effects on chronic diseases, including obesity [1,2,3,6].

### 2.1. Observational Epidemiological Studies of Anti-Obesity Effects of Green Tea/EGCG

Several observational human studies have indicated that tea or green tea consumption can exert beneficial effects on obesity. For example, a cross-sectional survey of 1210 epidemiologically sampled adults (569 men and 641 women) demonstrated that habitual tea drinkers (455 individuals were green or oolong tea consumers, and 18 were black tea consumers) for >10 years showed a 19.6% reduction in percent body fat and a 2.1% reduction in waist-to-hip ratios when compared with non-habitual tea drinkers [8].

Recent data from an epidemiological study with 6472 adult participants found that tea consumers had a lower mean waist circumference (WC) and lower body mass index (BMI) (25 vs. 28 kg/m^2^ in men; 26 vs. 29 kg/m^2^ in women) than non-consumers [1].

However, a recent study on 3539 participants observed that green tea was not associated with visceral obesity or metabolic syndrome (MetS) [9]. Thus, further studies are required to elucidate the anti-obesity properties of green tea.

### 2.2. Human Intervention Studies on the Effects of Green Tea/EGCG on Obesity

Several intervention studies have reported the beneficial effects of green tea [10,11]. In a randomized controlled trial (RCT) on 35 subjects with obesity and MetS, patients were randomly assigned to a control (four cups of water/day), green tea (four cups/day), or green tea extract (GTE) (two capsules and four cups of water/day) group for 8 weeks [12]. As per the findings of this study, it showed that green tea and GTE significantly decreased body weight and BMI vs. controls at 8 weeks (−2.5 ± 0.7 kg and −1.9 ± 0.6, respectively). These results further indicated the therapeutic effects of green tea catechins (GTCs) in improving MetS characteristics in obese patients.

A double-blind parallel multicenter trial in 240 subjects by Nagao et al. [13] observed that participants who consumed a GTC 583 mg/day dose exhibited greater decreases in body weight, BMI, body fat ratio, body fat mass, WC, hip circumference, visceral fat area (VFA), and subcutaneous fat area when compared to those who consumed a GTC 96 mg/day dose.

The anti-obesity benefits of green tea were also demonstrated in a meta-analysis of 24 human studies [14]. In total, 5 of the 11 trials in Eastern populations showed significant weight loss, ranging from 1 kg to 2 kg, and 3 of the 13 trials also demonstrated notable weight loss ranging from 1 kg to 9 kg.

In another RCT, 102 obese women were randomly divided into 2 groups: high-dose green tea group (EGCG, 856.8 mg/day) and the placebo group. The results indicated that 12 weeks of high-dose green tea resulted in significant weight loss (76.8 ± 11.3 kg–75.7 ± 11.5 kg) and decreased BMI, WC, total cholesterol (TC) and plasma low-density lipoprotein (LDL) levels, without any adverse effects to participants [15].

In another RCT, 73 obese women were divided into two groups: group A received GTE supplements for the first 6 weeks, while group B received daily placebo. After 6 weeks of treatment and a 14-day washout period, groups A and B were switched to placebo treatment and GTE treatment mutually for 6 weeks. These results showed that GTE effectively increased leptin and reduced LDL levels in these women [16].

Individuals with Down syndrome traditionally experience higher obesity rates [17]. In a double-blind phase II clinical trial in 77 young adults with Down syndrome, the placebo group showed increased body weight and BMI, which were not noted in an EGCG-treated group. The effect of EGCG on body composition was mainly observed in males, with significant differences between EGCG and placebo groups after 12 months, for body weight (estimated adjusted mean difference (AMD): −2.34, 95% confidence interval (CI): −4.21, −0.48) and body fat (AMD: −1.23, CI: −2.43, −0.04), suggesting that EGCGs were beneficial for weight management in patients with Down syndrome [18].

A recent systematic review and meta-analysis revealed that green tea supplementation had favorable effects on body weight (weighted mean difference (WMD): −1.78 kg, CI: −2.80, −0.75) and BMI (WMD: −0.65 kg/m^2^, CI: −1.04, −0.25). The reduction in WC after green tea consumption was determined to be significant for subjects using green tea at ≥800 mg/day (WMD: −2.06 cm) and treatment durations of <12 weeks (WMD: −2.39 cm). This dose-response observation indicated that green tea intake at <500 mg/day may reduce body weight over 12 weeks of treatment [19].

In contrast, several clinical trials failed to show body weight reduction via green tea consumption. A clinical trial in 151 participants aged 30–70 years showed that 1.8 g/day GTE consumption for 12 weeks did not lower body weight when compared with placebo, although a significant LDL-cholesterol (LDL-C) lowering effect was noted [20]. Data from an RCT of 937 healthy postmenopausal women who received decaffeinated GTE containing 843 mg EGCG reported that GTE was not associated with reductions in terms of body weight, BMI, or WC; further, it did not alter energy intake or mean hormone concentrations for over 12 months [21].

### 2.3. Laboratory Studies and Mechanisms of EGCG Action

Obesity is often characterized by the excessive accumulation of triglycerides (TGs), which are hepatically synthesized from both fatty acids (FAs) and de novo lipogenesis. Fatty acid synthase (FASN) is a central enzyme in lipogenesis and is responsible for FA production. The enzyme has gained considerable attention as a potential therapeutic target for obesity and cancer [22,23]. Zhang et al. [24] found that GTE inhibited FASN activity from duck liver, with a half-maximal inhibitory concentration (IC_50_) of 12.2 mg dry weight/mL [25]. Similarly, Tian et al. reported that EGCG inhibited FASN, with an IC_50_ of 52 μM, mainly via interactions with the FASN β-ketoacyl reductase domain.

Using a high-fat diet (HFD)-induced mouse obesity model, Wolfram et al. [26] observed that 1% (*w*/*w*) TEAVIGO, which contains 97.69% EGCG, prevented HFD-induced increases in mouse body weight. The authors also showed that FASN and acetyl-CoA carboxylase (ACC) mRNA levels were markedly decreased in adipose tissue. ACC has been identified as a key lipogenesis enzyme [27] and is potentially an anti-obesity target [28].

Both FASN and ACC expressions have been positively regulated by upstream lipogenesis transcription factors, including CCAAT/enhancer-binding proteins (C/EBPs) [29], peroxisome proliferation-activated receptors (PPARs, especially PPARγ) [30], and sterol regulatory element-binding proteins (SREBPs) [31]. Lee et al. demonstrated that 0.2% or 0.5% (*w*/*w*) EGCG can reduce body weight and the mass of several adipose tissues in a dose-dependent manner in an HFD-induced mouse obesity model [32]. These results indicated that EGCG lowered plasma TG and liver lipid levels. In the epidermal white adipose tissue of EGCG-treated mice, C/EBP, PPARγ, and SREBP mRNA levels were observed to be significantly decreased [32].

**Figure 2:**
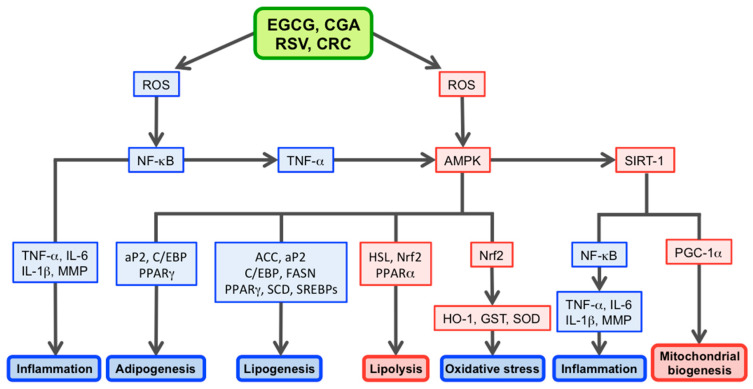
Potential anti-obesity mechanisms whereby EGCG, CGA, RSV, and CRC exert anti-obesity effects by scavenging/downregulating ROS. Red boxes represent upregulation/stimulation, whereas blue boxes represent downregulation/suppression.

5′-AMP-activated protein kinase (AMPK) has been identified as a metabolite-sensing protein kinase. Activated AMPK triggers beneficial physiological effects, including reductions in fat deposition. Ha et al. revealed that AMPK knockdown has upregulated fat-forming enzymes, including FASN, ACC, and stearoyl-CoA desaturase, which is another key enzyme of fat synthesis. These results suggest that activated AMPK inhibited fat synthesis by inhibiting lipogenesis [33]. Given that GTE and EGCG activated AMPK by inducing reactive oxygen species (ROS) generation [34,35], GTE and EGCG could improve body weight and lipid metabolism by activating AMPK through ROS generation. EGCG actions against lipogenesis and adipogenesis are shown in Figure 2.

Increased lipolysis can hydrolyze TGs and release free FAs, leading to anti-obesity effects. Treatment of pre-adipocyte 3T3-L1 cells with 10 μM EGCG for 24 h has been found to decrease intracellular lipid accumulation. Under the same experimental conditions, increased glycerol levels in the medium were observed, and hormone-sensitive lipase (HSL) mRNA levels, which catalyze rate-limiting stages in the hydrolysis of stored TGs to monoacylglycerol and free FAs, were similarly increased [36].

Consistent with these findings, Wistar rats on an obesity induced 8-week cafeteria diet, and supplemented with GTE (500 mg/kg body weight at 5 days/week for 12 weeks), led to significant reductions in obesity indicators, e.g., hyperlipidemia, fat synthesis, body weight, and fat depots when compared with the control diet group. Importantly, repression of *de novo* lipogenesis in adipose tissue, reduced lipid droplets in the liver, and insulin resistance development in diet-induced obese rats were accompanied by AMPK activation [37]. Similarly, mice fed a HFD with EGCG (50 and 100 mg/kg/day) exhibited significantly increased HSL mRNA expression in white adipose tissue when compared with the HFD group, and AMPK activity was noted to increase in both subcutaneous and epididymal adipose tissues in these mice [38]. These results suggest that GTE/EGCG decreased obesity in rats and mice via AMPK activation (Figure 2).

A recent study examining EGCG effects and mechanisms on lifespan extensions in obese rats demonstrated that median lifespans in control, HFD, and HFD + EGCG animals were 693, 599, and 683 days, respectively, indicating EGCG can restore shortened lifespans mediated by HFD [39]. EGCG also reduced inflammation and oxidative stress associated with aging in HFD-induced rats. EGCG significantly decreased blood circulating interleukin (IL)-6, tumor necrosis factor-α (TNF)-α, ROS, and superoxide dismutase (SOD) levels. EGCG also increased the expression of sirtuin-1 (SIRT1), catalase, fatty acid-binding protein-1, glutathione S-transferase (GST)-A2, and acyl-CoA synthetase-1, but significantly decreased nuclear factor (NF)-κB, ACC-1, and FASN expressions at the liver. These findings are represented by molecular events in Figure 2.

It has been suggested that GTE regulates β-oxidation enzymes, which are one of the indices for FA burning promotion and are activated by PPARα signaling [40,41]. Sae-Tan et al. [42] demonstrated that 0.32% dietary EGCG can reduce the body weight of HFD-induced mice, causing a 1.4–1.9-fold increase in PPARα mRNA levels when compared to HFD controls.

In mice fed with diets containing either low fat (5% TG), high fat (30% TG), or high fat supplemented with 0.1%–0.5% (*w*/*w*) GTC for 11 months, Murase et al. [43] observed that GTC significantly reduced HFD-induced body weight gains and visceral and liver fat accumulation and prompted the development of hyperinsulinemia and hyperleptinemia. GTC supplementation for 1 month increased hepatic mRNA expression levels of acyl-CoA oxidase and medium chain acyl-CoA dehydrogenase, as well as β-oxidation activity.

It is also possible that EGCG may have exerted its anti-obesity effects by reducing lipid uptake and transport. The multi-ligand receptor, cluster of differentiation 36 (CD36), has been determined as a key transmembrane protein implicated in lipid uptake and transport [44]. EGCG decreased TCs and TGs in mice given 1,3-dichloro-2-propanol (1 mg/kg body weight/day), which is an inducer of oxidative stress. EGCG dramatically increased the expression of phosphorylated AMPK at Thr172 and lowered CD36 expression [45]. CD36 has also been deemed essential for lipoprotein binding at the liver [46]. Therefore, decreased CD36 expression induced by EGCG may be related to changes in blood lipid profiles [45]. Thus, EGCG inhibition of CD36 via AMPK activation may contribute to its anti-obesity effects [47].

## 3. The Effects of Coffee/CGA on Obesity

Coffee and CGA have been determined to exert anti-obesity effects in humans [48], with several observational and experimental epidemiological studies supporting this hypothesis.

### 3.1. Epidemiological Studies on Coffee/CGA

A cross-sectional study in 137 patients with non-alcoholic fatty liver disease (NAFLD) and 108 controls showed that coffee consumption was inversely associated with obesity and insulin resistance [1,49]. The results of a Mendelian randomization study in 93,179 individuals demonstrated that coffee intake of up to four cups/day was associated with a lower risk of obesity with an odds ratios (ORs) of 0.82–0.86, when compared with non-coffee drinkers [50].

Yonekura et al. [51] conducted a cross-sectional study in 232 Japanese women aged 40–65 years and found that daily coffee consumption was inversely associated with high BMI (adjusted OR: 0.14, CI: 0.14, 0.96) and body fat percentage (adjusted OR: 0.33, CI: 0.14, 0.82).

Recently, Koyama et al. [9] reported that coffee consumption is associated with significantly lower levels of visceral obesity (OR: 0.746, CI: 0.588, 0.947) and MetS (OR: 0.706, CI: 0.565, 0.882). These findings indicate the beneficial effects of coffee consumption toward obesity. 

However, several studies failed to show such favorable effects. For example, an epidemiological study in 17,953 Korean adults aged 19–65 years failed to show that coffee consumption has health benefits; the OR for obesity of those who drank coffee ≥3 times/day was 1.37 (CI: 1.15, 1.63) when compared with those who had coffee <1 time/week [52]. Similarly, an epidemiological study in 5995 women indicated that the ORs of high coffee consumption (≥3 cups) were positively associated with obesity as measured by BMI (OR: 2.52, CI: 1.91, 3.34) and abdominal obesity as measured by WC (OR: 2.11, CI: 1.59, 2.79) when compared with non-coffee drinkers [53]. Coffee with additives such as sugar was positively correlated with the prevalence of obesity.

### 3.2. Clinical Studies on Coffee/CGA

A systemic review of three RCTs, including 142 participants, observed a significant difference in body weight in a green coffee extract (GCE)-consuming group when compared with placebo (mean difference: −2.47 kg, CI: −4.23, −0.72) [54].

A 12-week RCT in 30 overweight individuals reported that the average body weight loss in subjects who consumed CGA-enriched coffee was 5.4 kg, while in the instant coffee groups, this was 1.7 kg, suggesting a beneficial effect of CGA on body weight management [55].

Haidari et al. [56] conducted an 8-week RCT where 64 obese women were divided into the intervention group (receiving 400 mg GCE, equivalent to 180 mg CGA, *n* = 30) or the control placebo group (receiving 400 mg starch, *n* = 34). As per the study results, significant reductions were noted in body weight, BMI, and fat mass indices, and waist-to-hip circumference ratios in both groups; however, the decrease was higher in the intervention group. In addition, serum TC, LDL, leptin, and plasma free FA levels were significantly decreased in the intervention group, after adjusting for energy and fiber intake.

In another RCT, 142 healthy overweight men and women were divided into two groups: the high CGA group (369 mg CGA/serving) and the control coffee group (35 mg CGA/serving). Coffee was consumed once daily for 12 weeks, with 4-week pre- and post-observation periods. Body weight, BMI, VFA, total abdominal fat area (TFA), and WC were all significantly decreased in the high CGA group when compared with the control group. Changes in VFA (−9.0 ± 13.9 cm^2^ in the CGA group vs. −1.0 ± 14.3 cm^2^ in the control) and TFA (−13.8 ± 22.9 cm^2^ in the CGA group vs. −2.0 ± 16.2 cm^2^ in the control) from baseline to 12 weeks were found to be significantly higher in the high CGA group than in the control group [57].

An 8-week RCT was conducted in patients with NAFLD; patients were divided into an intervention group (400 mg green GCE containing 100 mg CGA, *n* = 24) or a placebo group (*n* = 24). The authors observed that GCE supplementation significantly reduced BMI (mean difference (MD): −0.57, CI: −0.84, −0.29) and increased serum high-density lipoprotein cholesterol (HDL-C) levels (MD: 7.06, CI: 0.25, 13.87) when compared with the placebo group. Serum TC decreased significantly in the GCE group (MD: −13.33, CI: −26.04, −0.61) [58].

However, an RCT in 18 healthy male subjects who consumed 185 mL of a test beverage with or without CGAs (329 mg) per day for 4 weeks showed no effects on body weight, BMI, or body fat, although a significantly higher postprandial energy expenditure was observed in the CGA group when compared with the controls [59]. Thus, further studies are warranted to determine the effects of coffee and CGAs on obesity.

### 3.3. Laboratory Studies and Mechanisms of CGA Action

Several basic studies have generated evidence to support the beneficial effects of CGA on obesity. Cho et al. demonstrated that 0.02% (*w*/*w*) CGA supplementation can lower body weight and TG levels in plasma, liver, and heart in a HFD-induced mouse obesity model [60]. These authors also showed that CGA significantly inhibited liver-based FASN when compared with the high-fat group. In accordance with these findings, Huang et al. observed that 90 mg/kg CGA consumption suppressed HFD-induced increases in body weight, and downregulated FASN and ACC mRNA expression in rats [61].

In a rat study, CGA supplementation (~100 mg/kg/day) reduced inflammation and fat deposition in the liver, along with reduced plasma liver enzyme activities in diet-induced obese rats [62]. The inhibitory effects of fat-forming enzymes by CGA may be in part due to the regulation of upstream lipogenesis transcription factors. Wang et al. demonstrated that mice fed a HFD with CGA (150 mg/kg) had significantly lowered body weight in comparison to HFD-fed control mice. In epididymal adipose tissue, CGA significantly decreased FASN expression and expression of the upstream transcription factors, C/EBP, PPARγ, and SREBP, but it increased PPARα expression [63]. These findings agreed with the previous HFD-induced obesity studies in murine models [61,64], suggesting CGA improved metabolic homeostasis.

Additionally, CGA may reduce lipogenesis via AMPK activation, thereby inhibiting fat-forming enzymes [65]. In HepG2 cells, CGA (0.5–10 mM) induced AMPK activation in a dose- and time-dependent manner (0.5–24 h). Activated AMPK then inhibited fat synthesis by inhibiting fat-forming enzymes and lipogenesis transcription factors [33]. Hou et al. also demonstrated that CGA-treatment (250 μM and 1000 μM) for 24 h can induce ROS production in human colon cancer cells (HCT116 and HT26) [66]. Since AMPK was activated by ROS [67], CGA could regulate lipid metabolism via AMPK activation through ROS generation. Figure 2 outlines several CGA actions.

The ability of CGA to increase lipolysis may be associated with its anti-obesity effects. Flanagan et al. examined the long-term health benefits of CGA. They observed that CGA consumption for 192 h increased lipolysis, as measured by free FAs and glycerol [68]. In 3T3-L1 cells, 20 μM CGA increased lipolysis by upregulating HSL expression [69,70]. These results suggested that CGA promoted lipid digestion by upregulating lipase expression.

Xu et al. [71] reported that mice fed a HFD supplemented with CGA and caffeine had significantly increased HSL and AMPK mRNA expression in the liver, when compared with the HFD group. CGA may also upregulate enzymes involved in FA β-oxidation, which in turn facilitates hepatic lipid degradation by activating PPARα in the liver and adipose tissue [72]. These results indicated that CGA decreased obesity by enhancing lipolysis via AMPK activation [71] (Figure 2).

Ma et al. [64] reported that mice fed a HFD supplemented with CGA (100 mg/kg, twice a week) significantly suppressed the hepatic expression of CD36 when compared to the HFD group. Given that AMPK signaling plays a key role in CD36-induced lipid absorption [47], CGA may inhibit CD36 via AMPK activation, leading to reduced lipid uptake and transport. CGA may also affect lipid digestion by suppressing bile acid function, which is deemed critical for the digestion and absorption of lipids at the small intestine.

The microRNA-122 (miR-122) is liver specific and plays a critical role in liver homeostasis [73]; its inhibition is associated with the gene suppression of key roles in liver lipid metabolism, such as FASN [74]. Murase et al. demonstrated that CGA increased miR-122 levels in Hepa1–6 cells [75]. These results suggested that CGA may inhibit lipogenesis through post-transcriptional mechanisms.

## 4. The Effects of Wine/RSV on Obesity

### 4.1. Observational Epidemiological Studies on the Anti-Obesity Effects of Wine/RSV

Epidemiological studies have suggested that light to moderate consumption of alcohol may have protective effects against obesity [1]. A prospective cohort study which enrolled 15,920 normal-weight (BMI: 18.5 kg/m^2^ to <25 kg/m^2^) postmenopausal women observed that the risk of becoming overweight and obese over a 7-year follow-up period was 35% and 88% lower, respectively, for women in the upper quintile of alcohol intake, relative to non-drinkers [76]. Of the alcoholic drinks, wine consumption showed the greatest inverse association for the risk of being overweight (hazard ratio (HR): 0.75, CI, 0.68, 0.84), followed by liquor (HR: 0.85, CI: 0.78, 0.93) and beer (HR: 0.90, CI: 0.82, 1.00). Vidot et al. [77] reported that among wine drinkers, low and moderate drinkers had a lower OR for MetS when compared with non-drinkers (OR: 0.72, CI: 0.55, 0.96 and OR: 0.43, CI: 0.21, 0.87, respectively). However, no significant associations were found for heavy wine (OR: 1.16, CI: 0.43, 3.16) and liquor drinkers.

Inan-Eroglu et al. [78] examined the associations between types of alcoholic drinks and adiposity in a large United Kingdom cohort (*n* = 280,183, 48.3% female). Study data indicated that when compared to non-wine drinkers, red wine, champagne, and fortified wine drinkers had lower BMIs (differences were as follows: −0.75 kg/m^2^, CI: −0.78, −0.72 kg/m^2^; −0.48 kg/m^2^, CI: −0.52, −0.45 kg/m^2^; and −0.24 kg/m^2^, CI: −0.29, −0.18 kg/m^2^, respectively). Beer and spirits drinkers were determined to have higher BMIs when compared with non-beer and spirit drinkers (difference: 0.18 kg/m^2^, CI: 0.14, 0.22 kg/m^2^, and difference: 0.64 kg/m^2^, CI: 0.61, 0.68 kg/m^2^, respectively). A population-based study by Osella et al. showed that >10 g/day of wine consumption was associated with being overweight [79].

A cross-sectional study in healthy volunteers (1481 women aged 35–60 years and 1210 men aged 45–60 years) showed a J-shaped relationship of waist-to-hip ratios and BMIs, in terms of wine consumption [80]. When compared with non-drinkers, men consuming <100 g/day wine had a lower BMI, and lower waist-to-hip ratio was found in both men and women.

These findings suggest that low and moderate wine consumption may exert beneficial effects on obesity. However, a follow-up survey of 8103 subjects with a mean age of 35.4 years during ≥6 years failed to show such beneficial effects; instead, results showed a non-significant association between wine consumption and MetS [81].

### 4.2. Human Intervention Studies Investigating the Effects of Wine/RSV on Obesity

Intervention studies have reported on the obesity-related effects of wine consumption. In an RCT in obese subjects who habitually consumed moderate alcohol levels, 40 of 49 eligible participants (BMI, 34.2 ± 6.4 kg/m^2^) completed the 3-month intervention study on a 1500 kcal dietary regimen, one with 10% of energy from white wine and one with 10% of energy from grape juice [82]. These results showed that all subjects achieved significant body weight reduction; percent body fat, WC, blood pressure, blood glucose, insulin, TGs, and cholesterol were also reduced. In contrast, another RCT revealed that moderate wine consumption in overweight women did not improve insulin sensitivity and any other correlates of insulin sensitivity, i.e., body weight, blood lipids, and blood pressure [83].

In contrast to the studies focusing on wine consumption, the potential health benefits of RSV consumption have also been examined. Following a literature search, Wang et al. [4] summarized five RCTs published between 2009 and 2013, of which four reported favorable RSV effects. These included the downregulation of inflammatory markers, such as TNF-α and plasminogen activator inhibitor-1 (three studies), TGs (one study), LDL-C (one study), and nuclear factor-κB (NF-κB, one study), and the upregulation of adiponectin (two studies), AMPK (one study), and SIRT1 (one study) [4].

In a comprehensive clinical study review, Wahab et al. [84] discussed the effects of RSV, and included 17 studies in patients with chronic diseases and 21 studies in healthy subjects. These authors reported the beneficial effects of RSV, including reduced body weight, BMI, fat weight, LDL-C levels, and adipocyte size, in addition to the downregulation of inflammatory cytokines such as TNF-α. The authors also recorded elevated adiponectin levels and adipose tissue lipolysis.

In a recent review, Singh et al. [85] summarized a number of RSV clinical trials in patients with obesity, diabetes, cancers, MetS, Alzheimer’s disease, cardiovascular disease, and inflammatory disease. These authors observed that RSVs exerted favorable effects in 13 of the 15 studies in patients with obesity, being overweight, and MetS.

In contrast, several studies published conflicting results in this area. For example, a study by Poulsen et al. [86] showed that the consumption of RSV (500 mg for 28 days) by 24 obese, but otherwise healthy males, resulted in non-significant changes in terms of obesity markers, total body mass, total body fat mass, and visceral and abdominal subcutaneous fat volumes. Meanwhile, Gualdoni et al. [87] reported that 10 healthy volunteers who consumed 5 g RSV showed a significant increase in TNF-α levels at 24 h after treatment when compared at baseline. Peripheral blood mononuclear cells or isolated monocytes showed that RSV potentiated TNF-α production stimulated by different Toll-like receptor agonists. Moreover, significant increases in NF-κB activities and p105 phosphorylation were indicative of alternative NF-κB pathway activation.

### 4.3. Laboratory Studies and RSV Mechanisms

Most cell-based and animal studies have described favorable effects for RSV, thereby supporting findings from epidemiological cohort and intervention studies. Baur et al. [88] demonstrated that RSV alleviated the negative impact of a high-calorie diet on overall health and lifespan in middle-aged mice. RSV increased insulin sensitivity, AMPK, and peroxisome proliferator-activated receptor-γ coactivator-1α (PGC-1α) activity and reduced insulin-like growth factor-1 levels. Parametric analyses revealed that RSV gave the significant opposed effects of the high-calorie diet in 144 out of 153 altered pathways. Although this study showed that increased SIRT1 enzymatic activity by RSV without altering its gene expression, a different study showed that RSV restored its decreased mRNA levels induced by hemorrhagic shock in the rat kidney to the control levels [89].

Price et al. [90] reported different RSV dose-dependent activities toward AMPK and SIRT1 expression. Mice treated with a moderate RSV dose were able to activate AMPK, increasing mitochondrial biogenesis and function in skeletal muscle, whereas SIRT1 knockout mice displayed none of these effects. A mouse model over-expressing SIRT1 mimicked these effects, demonstrating SIRT1 was required for AMPK activation. In contrast, a high RSV dose activated AMPK in a SIRT1-independent manner, demonstrating RSV dosage was a critical factor [90]. These findings indicated that RSV exerted dose-dependent effects on AMPK and SIRT1 signaling pathways.

Wang et al. [4] reviewed 14 cell and 12 animal studies investigating the anti-obesity effects of RSV. Their data indicated: RSV increased adiponectin levels; reduced TG levels and lipid accumulation; upregulated AMPK, SIRT1, and SIRT3; downregulated FASN and PPARγ; ameliorated drug-induced increases in TNF-α production; and activated NF-κB. Animal study data indicated that the anti-obesity effects of RSV in animals were potentially mediated through stimulation of fat oxidation and metabolism, together with the suppression of adipogenic gene expression, such as PPAR, C/EBPα, SREBP-1c, FASN, lipopolysaccharide (LPS), FA-binding protein (adipocyte Protein 2 (aP2)), and leptin. They also indicated that RSV not only downregulated obesity-induced chronic inflammation expression of TNF-α, interferon (IFN)-γ, IFN-β, and IL-6, downstream signaling molecules, and oxidative stress, but it also upregulated anti-oxidant defense capacities, such as increased liver SOD, glutathione peroxidase, and catalase.

Based on clinical and preclinical data, Singh et al. [85] proposed the following RSV molecular targets: AMPK, GST, IL-1β, matrix metalloproteinase (MMP), NF-κB, nuclear factor erythroid like 2 (Nrf2), PGC-1α, SIRT1, and TNF-α. Possible mechanisms, involving these targets, whereby RSV exerts anti-obesity effects are shown in Figure 2.

## 5. The Effects of Curry/CRC on Obesity

### 5.1. Human Studies on Curry/CRC

No comprehensive epidemiological studies have yet reported the effects of CRC on obesity. Future observational human studies may reveal its beneficial effects on obesity and other diseases, particularly in Asian populations which have high CRC consumption levels.

However, multiple clinical studies have reported on the health benefits of CRC consumption. In an RCT in MetS patients with BMIs between 25.0 and 29.9, 44 participants showed a <2% weight loss after 30 days of diet, 22 patients were treated for a further 30 days with a CRC mixture (800 mg/dose/day of *Curcuma longa* extract containing 95% CRC), while the remaining 22 patients received vehicle (400 mg/dose/day of pure phosphatidylserine) [91]. These results indicated that CRC treatment increased weight loss from 1.88% to 4.91%, enhanced the percentage reduction of body fat (0.70% to 8.43%), waistline reduction (2.36% to 4.14%), hip circumference reduction (0.74% to 2.51%), and BMI reduction (2.10% to 6.43%) [91].

Data from four clinical studies led Kunnumakkara et al. [92] to conclude that CRC was effective in reducing anxiety and depression symptoms associated with obesity. CRC modulated circulating IL-1β, IL-4, and vascular endothelial growth factor (VEGF) levels to generate immunosuppressive effects and reduce oxidative stress in obese patients.

A systematic review of seven RCTs examining turmeric and CRC in patients at risk of cardiovascular disease suggested they had beneficial effects on serum TG and LDL-C levels, although no significant differences were found for serum HDL-C levels. When the analysis was restricted to more homogenous studies based on underlying different disease categories, a beneficial effect of turmeric and CRC on serum TC levels was noted in patients with MetS [93].

A meta-analysis of eight RCT reported a significant reduction (WMD: −4.69 pg/mL, CI: −7.10, −2.28) in circulating TNF-α levels upon CRC supplementation, suggesting a beneficial effect of CRC on inflammation [94].

A meta-analysis of 11 studies involving 876 subjects (53% women) found a significant effect of CRC on body weight (WMD: −1.14 kg, CI: −2.16, −0.12) and BMI (WMD: −0.48 kg/m^2^, CI: −0.78, −0.17), respectively [95].

In an RCT by Saraf-Bank et al. [96], 60 overweight and obese adolescent girls were randomly assigned to either a placebo or CRC intervention group. Supplementation with 500 mg/day CRC for 10 weeks was found to significantly lower IL-6 levels and oxidative stress markers, suggesting beneficial effects on inflammation and oxidative stress.

However, data from a 6-month RCT in elderly subjects who consumed CRC or placebo showed that CRC consumption (doses of either 1 g or 4 g/day) did not significantly affect TG, TC, LDL-C, or HDL-C levels over 1 or 6 months [97]. Data from another RCT, where 30 obese individuals were randomized to receive 1 g/day CRC or placebo, showed that CRC significantly reduced IL-1β, IL-4, and VEGF serum levels, but no significant differences were observed for IL-2, IL-6, IL-8, IL-10, IFN-γ, epidermal growth factor, and monocyte chemoattractant protein-1 levels [98]. Thus, different studies show that the CRC effects on some biomarkers are variable.

### 5.2. Laboratory Studies and CRC Mechanisms

Several cell and animal studies have demonstrated the beneficial effects of CRC on obesity [4]. In a mouse model study, CRC administration for 28 weeks significantly attenuated the effects of HFD on body weight gain, glucose disposal, and insulin resistance. CRC also inhibited the expression of lipogenic genes, including NF-κB, SREBP-1c, and carbohydrate-responsive element-binding protein in the liver, and blocked the effects of HFD on inflammatory pathways in adipose tissue [99]. Similarly, dietary CRC ameliorated diabetes in HFD-induced obese and *ob/ob* male C57BL/6J mice and further reduced body weight gain. CRC also decreased macrophage infiltration in white adipose tissue, adipose tissue adiponectin production, hepatic NF-κB activity, and hepatic inflammation [100].

In 3T3-L1 cells, Ahn et al. [101] showed that CRC decreased aP2 (a mature adipocyte marker) mRNA expression, increased c-Myc and cyclin D1 expression (well-known Wnt targets), and inhibited mitogen-activated protein kinase (MAPK) phosphorylation, which has been associated with 3T3-L1 differentiation into adipocytes. These findings suggest that the Wnt signaling pathway participated in CRC-induced suppression of adipogenesis in 3T3-L1 cells.

Wnt signaling activation represses adipogenic differentiation. The transcription factor 7-like 2 (*Tcf7l2*) gene encodes a key Wnt signaling pathway effector, and its human homologue, *TCF7L2*, may be a high-risk gene for diabetes. Tian et al. [102] showed that CRC attenuated miR-17-5p expression and stimulated *Tcf7l2* expression in 3T3-L1 cells. Since miR-17-5p expression in mouse epididymal fat tissues increased in response to HFD, these finding suggested that miR-17-5p could be a central switch in adipogenic differentiation.

Ejaz et al. [103] demonstrated that in mice fed HFD, CRC reduced body weight gain, adiposity, and micro-vessel density in adipose tissue, which coincided with the reduced expression of VEGF and its receptor, VEGFR-2. CRC has also been noted to activate AMPK, reduce glycerol-3-phosphate acyl transferase-1, and increase carnitine palmitoyltransferase-1 expression, leading to increased lipid oxidation and decreased fatty acid esterification.

Kunnumakkara et al. [92] characterized CRC molecular targets, including transcription factors, protein kinases, inflammatory mediators, apoptotic regulators, protein reductases and histone acetyl transferase, growth factors, receptors, and adhesion molecules. CRC may exert its multiple effects via epigenetic regulation, of which the major targets include Nrf2, β-catenin, NF-κB, p38 MAPK, cyclooxygenase-2, forkhead box O3, inducible nitric oxide synthase, ROS, cyclin D1, VEGF, glutathione, TNF-α, and extracellular-regulated protein kinase.

Wang et al. [4] reviewed 11 cell-based, 16 animal, and 3 human studies investigating the anti-obesity effects induced by CRC and concluded CRC could down- or upregulate various transcription factors, enzymes, cytokines, and other signaling pathway components. More specifically, downregulated or inactivated molecules included C/EBP-α, PPARγ, SREBP-1c, FASN, ACC, HSL, SREBP-1c, NF-κB, IL-6, and TNF-α. Those upregulated or activated included AMPK, Nrf2, and SIRT1. How these molecules are involved in CRC anti-obesity effects is highlighted in Figure 2.

## 6. Discussion

This review summarized the favorable anti-obesity effects of consuming green tea, coffee, wine, and curry and their principal associated polyphenols. While considerable human observational and intervention studies have expounded this hypothesis, several studies have failed to show any beneficial effects. Such differences may have been due to confounding factors, e.g., differences in study design, quantifying consumption methods, beverage temperatures, cigarette smoking, alcohol consumption, and differences in genetic and environmental factors, such as race, sex, age, lifestyle, intestinal microbiota, and genetic polymorphisms [2,104,105]. Further complicating these issues is the fact that polyphenols have limited bioavailability as their absorption from the human digestive system is restricted; thus, they are predominantly metabolized in the gut and liver [106]. In the future, more comprehensive and definitive human studies should be performed to assess the bioavailability of polyphenols and prove the anti-obesity effects of consuming these foods.

Most animal and cell-based studies supported the beneficial findings of human studies. These studies also proposed possible mechanisms underlying the anti-obesity actions of these polyphenols. Figure 2, which has been generated from previous data [1,3,6,33,85,105,107,108,109] illustrates putative mechanism showing how EGCG, CGA, RSV, and CRC may exert their anti-obesity effects. These mechanisms are underpinned by the downregulation/inhibition of adipogenesis, lipogenesis, oxidative stress, and inflammation and the upregulation/stimulation of lipolysis and mitochondrial biogenesis.

Polyphenols are frequently documented with dual pro- and anti-oxidant roles (Table 1). This table shows that EGCG, CGA, RSV, and CRC upregulate AMPK by stimulating ROS generation, but downregulates NF-κB by scavenging ROS. A caveat to this table is that not all studies are necessarily related to obesity. Currently, it remains unclear what factor(s) direct these polyphenols to act as pro- or antioxidants; however, differences in cellular polyphenol and metal ion concentrations, cell types, and the co-existence of other anti-oxidants may be important factors. Thus, further studies will be required to clarify these issues. It should be noted that AMPK activation via TNF-α downregulation (Figure 2) is based on Steinberg et al. [107]; however, more evidence is required for this observation.

Epigenetic modifications, including post-translational changes caused by miRNA dysregulation (miRNAs are small single-stranded molecules comprising 20 to 25 nucleotides), are implicated in various cellular processes via gene expression regulation [110,111]. Several miRNAs are associated with obesity [112], e.g., miR-17-5p, miR-122, and miR-221.

As described earlier, miR-122 and miR-17-5p were implicated as CRC and CGA targets, respectively [75,102]. The effects of EGCG, CGA, RSV, and CRC on miRNAs were then compared (Table 2), although it remains unclear how these miRNAs, other than miR-122 and miR-17-5p, are associated with anti-obesity effects mediated by these polyphenols. Table 2 indicates the polyphenols reviewed here downregulated miR-17 and miR-21, but differences exist among them in modulation of miR-33, miR-122, miR-155, and miR-221, suggesting different mechanisms may function in their anti-obesity effects.

Future studies will clarify whether these polyphenols have similar or different modulatory effects on these miRNAs.

## Figures and Tables

**Table 1:** Studies showing EGCG, CGA, RSV, and CRC modulatory effects on ROS, AMPK, and NF-κB. Studies showing ROS and AMPK stimulation or upregulation are in red boxes, whereas studies showing ROS and NF-κB suppression or downregulation are in blue boxes.

Polyphenols	ROS	AMPK	ROS	NF-κB
	Stimulation/Upregulation	Stimulation/Upregulation	Suppression/Downregulation	Suppression/Downregulation
EGCG	Tsai et al. [113] Hsieh et al. [114] Liu et al. [115]	Tan et al. [116] Bae et al. [117] Ueda et al. [118]	Wada et al. [119] Qin et al. [120] Yi et al. [121]	Zhong et al. [122] Wang et al. [123] Reddy et al. [124]
CGA	Rakshit et al. [125] Yang et al. [126] Hou et al. [66]	Zhou et al. [127] Jang et al. [128] Tsai et al. [129]	Han et al. [130] Gong et al. [131] Kong et al. [132]	Bao et al. [133] Tian et al. [134] Fu et al. [135]
RSV	Posadino et al. [136] Chen et al. [137] Li et al. [138]	Vlavcheski et al. [139] Guo et al. [140] Wang et al. [141]	Giordo et al. [142] Ramdani et al. [143] Zhang et al. [144]	André et al. [145] Subedi et al. [146] Xian et al. [147]
CRC	Liang et al. [148] Yu et al. [149] Nakamae et al. [150]	Soltani et al. [151] Lu et al. [152] Yu et al. [153]	Sadeghi et al. [154] Ran et al. [155] Lin et al. [156]	Li et al. [157] Khan et al. [158] Zhou et al. [159]

**Table 2:** MiRNA modulation by EGCG, CGA, RSV, and CRC polyphenols. Arrows in red boxes (↑) and blue boxes (↓) represent upregulation and downregulation, respectively.

miRNA	Polyphenols	Modulation	Cell/Animal Model	Dose	References
**miR-17**	EGCG	↓	Human umbilical vein endothelial cells	50 mg/mL	[160]
	CGA	↓	Human hepatocellular carcinoma Huh7 cells and human small cell lung cancer NCI-H446 cells	25, 50 mM	[161]
	RSV	↓	Human breast cancer cell lines (Bcap37, MDA-MB-231)	6.25, 25 mM	[162]
	CRC	↓	Mouse embryonic fibroblast 3T3-L1 cells	2, 10 mM	[102]
**miR-21**	EGCG	↓	Rat model of chronic renal injury	200 mg/kg	[163]
	CGA	↓	CCl4-induced liver fibrosis rat model	15, 30, 60 mg/kg	[164]
	RSV	↓	Human pancreatic stellate cells	50 μM	[165]
	CRC	↓	Rat model of liver fibrosis	100 mg/kg	[166]
**miR-33**	EGCG	↓	Human hepatoma HepG2 cells	50 mM	[74]
	CGA	↓	Hypercholesterolemic rats model	75, 150, 300 mg/kg of *Lonicera caerulea*berry extract containing CGA	[167]
	RSV	↑	Human hepatoma HepG2 cells	50 mM	[74]
	CRC	↓	Human THP-1 macrophages	40 mM	[168]
**miR-122**	EGCG	↓	Human hepatoma HepG2 cells	50 mM	[74]
	CGA	↓	Hypercholesterolemic rats model	75, 150, 300 mg/kg of*L. caerulea* berry extract containing CGA	[167]
	RSV	↑	Human hepatoma HepG2 cells	50 mM	[74]
	CRC	↑	Bile duct ligation-induced fibrotic rats	100 mg/kg	[169]
**mir-155**	EGCG	↑	Human colon cancer cell lines (HCT-116, DLD-1)	50 mM	[170]
	CRC	↓	LPS-treated murine monocyte/macrophage RAW264.7 cells	31.25, 62.5 mM	[171]
	RSV	↑	Mouse embryonic fibroblast 3T3-L1 cells	25 mM	[172]
	CRC	↓	LPS-induced mouse model of inflammation	20 mg/kg	[173]
**miR-221**	EGCG	↑	Rat pheochromocytoma PC12 cells	50 mM	[163]
	RSV	↑	Human umbilical vein endothelial cells	50 mM	[174]
	CRC	↓	Human hepatoma HepG2 cells xenograft mouse model	100 mg/kg	[169]

## Data Availability

Not applicable.

## Abbreviations

ACC	Acetyl-CoA carboxylase
AMD	Adjusted mean difference
AMPK	5′-AMP-activated protein kinase
aP2	Adipocyte Protein 2
BMI	Body mass index
C/EBP	CCAAT/enhancer-binding proteins
CGA	Chlorogenic acid
CD36	Cluster of differentiation 36
CI	Confidence interval
CRC	Curcumin
EGCG	Epigallocatechin-3-*O*-gallate
FAs	Fatty acids
FAS/FASN	Fatty acid synthase
GST	Glutathione S-transferase
GCE	Green coffee extract
GTE	Green tea extract
HR	Hazard ratio
HFD	High-fat diet
HSL	Hormone-sensitive lipase
IC_50_	Half-maximal inhibitory concentration
IFN	Interferon
IL	Interleukin
LPS	Lipopolysaccharide
LXR	Liver X receptor
LDL	Low-density lipoprotein
MMP	Matrix metalloproteinase
MD	Mean difference
MetS	Metabolic syndrome
MAPK	Mitogen-activated protein kinase
MS	Multiple sclerosis
NAFLD	Non-alcoholic fatty liver disease
Nrf2	Nuclear factor erythroid 2-like 2
NF-κB	Nuclear factor-κB
ORs	Odds ratios
PGC-1α	Peroxisome proliferator-activated receptor-γ coactivator-1α
PPAR	Peroxisome proliferation-activated receptor
RCT	Randomized controlled trial
ROS	Reactive oxygen species
RSV	Resveratrol
SIRT	Sirtuin
SCD	Stearoyl-CoA desaturase
SOD	Superoxide dismutase
SREBP	Sterol regulatory element-binding proteins
TFA	Total abdominal fat area
TC	Total cholesterol
Tcf7l2	Transcription factor 7-like 2
TGs	Triglycerides
TNF	Tumor necrosis factor
VEGF	Vascular endothelial growth factor
VFA	Visceral fat area
WC	Waist circumference
WMD	Weighted mean difference
